# On the Compensatory Relations between the Faculties of Order and Memory

**Published:** 1857-10-01

**Authors:** A. F. Mayo


### Art. VI.—ON THE COMPENSATORY RELATIONS BETWEEN THE FACULTIES OF ORDER AND MEMORY.

BY A. F. MAYO, ESQ., BARRISTER-AT-LAW.

I THINK it may be shown that Order and Memory, regarded as separate and independent faculties of the mind, stand related to each other as compensating faculties; in other words, that a deficiency in either of these faculties can, in some degree, be supplied by the other faculty.

In the first place, I will inquire how the presence of Memory compensates for the absence of Order, both being regarded on this occasion as independent faculties of the mind.

Memory involves the power of retaining or fixing for permanent use the ideas which, from any cause, whether internal or external to the mind, occur to the mind. It may seem strange that Memory, which from the abundance of the wealth which it enables the mind to conserve and store up for future use, might seem, more than all the other faculties of the mind, to require the assistance of Order, steward-like, to arrange its accumulations, should have a facility of its own in dispensing with the assistance of Order. Yet that it is so, I think I shall show my reader.

The one great difficulty in giving harmony to the ideas which we derive from so many sources, arises from the associations and prejudices which cling to the ideas. Thus, what the ideas gain in bulk and thickness they lose in delicacy and clearness of outline. Each new idea which we form has, in proportion to its novelty, a cloud of associations attaching themselves to it, the result often of the casual circumstances of the moment at which it was impressed. These associations, while they tend to amplify the scope and dignity of the new idea which we have gained on one subject, in the same proportion tend to the disparagement of our former ideas on the same subject. Here the faculty of Order, if present in strength, would insist on showing the symmetry and bearing of our new ideas; in other words, would harmonize our new knowledge with our old ideas, and thus with our old knowledge.

But if the faculty of Order is weak, and that of Memory strong, the mind, instinctively tending to consolidate its footing, will summon instinctively Memory to its aid, which, under the mere laws of association, will bring to our consciousness hosts of old ideas, which will modify the new ones, and deprive them of their arrogant claim to enthrall the attention.

The old idea thus becoming associated with the new idea, will, by the aid of Comparison, immediately become the measure of the new idea; and thus also Causality will often exert its function to show the essential moving principle common both to our new and old ideas.

Nor to produce this effect need we suppose the faculties of Comparison and Causality strongly developed. For the brightness of the images which a strong Memory reproduces makes their critical function an easy one. Thus the mental idols (in the sense of Lord Bacon) which are stamped on the mind by the passions, the pursuits, and the other individualities of each man, and which so often intrude on the mind when excited by the orgasm of a novel conception, are, by the aid of Memory, coolly brought face to face with our antecedent knowledge, and often will retire abashed.

This effect is often produced by the merest juxtaposition, independent of any visible exercise of Comparison and Causality. In this way the new idea is controlled by the memory of past ones, and being divested of its superfluous attire, is ready for inscription in those mental tablets for the registration of particulars, on the basis of which alone all sound inquiries must be conducted. The new idea is like an obtrusive home-boy, who, placed in a public school, at once loses his offensive impertinence under the mere pressure of numbers, producing a constant abrasion of the edges of individual minds. The remembered ideas will also confer upon the new one the polish of uniformity, and if detracting in some respect from its picturesqueness, will give more than an equivalent in adding to its usefulness. Where, however, the power of memory is deficient, but is nevertheless invoked, a confused idea is the probable result, from which the thinker recoiling falls back upon his new idea. Otherwise the confusion in the recollection of former ideas is propagated into the new idea itself. Suppose a man to have presented to him a new tenet of morality, which chimes in with his prepossessions. If he cannot recollect any analogous cases except very feebly, he would do better to apply to the faculty of Order to test the general reasonableness of the doctrine, than to endanger it by specific yet untrustworthy analogies.

Minds with a strong passion for truth, and only choosing to recognise ideas which are distinct, will, in such a case, discard the incumbrance of imperfect recollections. Without the aid of Memory, the mind depending on mere association could never rely on its own capacity to summon up a group of past ideas which should be united together with any closeness of adhesion; hence, minds which have to deal with new ideas, in their carefulness not to impair their clear and vigorous reality by the shadowy forms of dimly remembered past ones, will, in their defect of Memory, be disposed to ignore, not simply their forgotten ideas, but all that general result of such forgotten ideas which express their sum and substance in the convictions of the present: while others, by reason of the same defect of Memory, habitually check the fine elasticity of their intellect, restraining it, as huntsmen do their greyhounds in a leash, in fear lest, in giving way to such elasticity of mind, they may compromise truths which are in fact forgotten, or mitigate their authority if they should be recalled to the mind in future. They do this from an instinctive apprehension that the possession of a mind by a new idea may make the retracing of a former idea on some subject so much the more difficult, unless it can be renewed by force of contrast. On the other hand, suppose that a man's memory is strong, and that he has under a flexible command a large army of acquired ideas and facts; we shall generally observe that he can place them in a rough sort of order, however destitute of method his mind may otherwise appear to be. The memory of a Lyndhurst or Macaulay will present them with visions so bright in colour, so clear in outline, so girt with all the moving lineaments of the life which they possessed when they were first mirrored from their object upon the mental sensorium, that the task of Order in allotting them a collocation will be small; and this on the same principle that while the most adroit captain called on to marshal his battalion in perfect darkness would assuredly fail, they would be nowhere, as are the ideas of the past to him who has forgotten them: but pour the light of day on the battalion, and then a school-girl might arrange them with some pretence of symmetry.

All large masses of ideas, like all large masses of men, necessitate by their very presence, a rough sort of Order to prevent them from jostling and crowding each other. The man in the crowd must have standing room. He must also adjust his position in some harmony with that of his neighbour. So an idea, by virtue of its being remembered, must have some clearness of outline, and in proportion to this *last*, which is made up partly of the strength of the impression and partly of the strength of the memory, has it a facility for coming into accordance with other ideas.

That the faculty of Order should compensate for the absence of Memory seems a corollary from the above remarks. I can make it more evident. The faculty of Order places ideas in certain synthetic relations to each other. These synthetic (universal) relations must have a connecting link. This link is obtained by the faculty of Order. Order therefore has a sympathy with the design or ultimate object of any course of action, and similarly with the general tendency of a collection of facts. It does not so much investigate their separate truth or qualities ; but, assuming these as proved, strives to obtain their general harmony and result. It performs the functions of a gardener who surrounds his younglings with a fence, or places them within an iron frame. Besides this, the faculty of Order provisionally tickets each new idea which it encounters according to certain preconceived generalizations. Thus, to the struggling infancy of the idea of which Comparison and Causality have been the inventors, Order offers a home and shelter under the bosom of recognised and admitted truth. In this way, Order defends the delicate speculations of the analytic faculties from a premature destruction. Those who do not recollect that without speculation unverified in the first instance the laws of no single science could ever have been discovered, love to nip in the bud the luciferous speculations of even undoubted genius. This perverse hatred of generalization and dislike of innovation which beset common minds, are soothed into neutrality by the intervention of the faculty of Order, when it squares and cunningly adapts that which is as yet an unverified speculation to an agreement with recognised laws. Yet does not this take place without danger from those anticipations of truth which the verification of after inquiry does not always confirm. If, however, the provisional generalizations are well remembered to be only tentative guesses at truth, to be subjected to the probation of inquiry, the gain is all on the side of truth.

We have seen that Memory recals former ideas in a freshness and beauty which is even purified by the lapse of time, and modifies our admiration for the most recent child of our thought by the juxtaposition of our former knowledge.

This result Order effects, but in an inverse manner. While the man of strong memory gives full swing to the associations caused by the idea before him, however prurient they may be, in order that the images of the past catching fire should appear in brightness, the man strongly gifted with Order lops off the associations which ivy-like conceal the symmetry of the new idea and its analogies with old ideas, or he packs it up in the shape in which it is most portable, and in which it may be most conveniently laid alongside of old ideas. With a man, then, of strong memory, the arrogance of the new idea is chastened by the recollection of ancient facts; with a man strongly endued with order it is chastened by a reference to established laws.

While Comparison may be said to estimate and observe the rough lineaments of phenomena, and the varieties and difference of the surface of things, and while Causality penetrates into the hidden essence of things, thus confining itself within a narrower area than Comparison, but digging to a greater depth, Order takes a further sweep than either into the expanse of Nature. Akin to Comparison in its love of extent of observation and to Causality in its love of boldness of theory, it carries both further than Comparison and Causality respectively. In its love for catching at large general laws and combinations, and for observing the mutual action of such laws and the general harmony of the universe, it stands alone. Its danger lies in its soaring spirit, in its despising the ground, when it can find symmetry and beauty in ideal realms. Its strength lies in the firmness of its alliance with the two sister faculties, Comparison and Causality, and while it plumes itself as being a pioneer in mental inquiry, as well as an ultimate refiner and purifier of mental wealth already acquired, it is bound not to forget those stern delvers in the search for knowledge, who dealing at first hand with Nature, sustain the heaviest burden. Order has a speculative bearing, in the extent of its telescopic sweep over the realm of nature. It has a practical bearing in its showing the co-operation in action of various coefficients.

Comparison and Causality, often too much occupied in an intense regard of objects, with a view to nothing further than their separate natures, forget occasionally as well their practical, as their theoretic value. I mention all this to indicate with what a strength of grasp the faculty of Order co-ordinates or views, in general relations, the facts and ideas of nature. It may be asked, in what way this faculty disposes of those superfluities of separate objects which it is compelled to elide that their facilities of combination may become visible? It does not throw them away; they, too, may be ticketed by Order, and provisionally bracketed with similar residuary facts.

Each new fact or idea may thus be made a probationary member of a system, from which however it must be displaced if further inquiry, by Comparison and Causality, should show its collocation to be founded in error. Even pure association, when the mind is not conscious of any exertion, but allows the panorama of the past to move silently before it, is often subjected to the latent influence of Order. The full explanation of the laws connecting Association with this faculty more than with the other faculties of the mind, I must reserve to another occasion. Even at first sight it cannot surprise us that the mental system should be largely indebted to the faculty of Order as a physcian to remedy the morbid action of Association. Frequently, without exciting our notice, the faculty of Order will step in among the associations, and while leaving them their outward form and gesture, will reduce the stragglers to some provisional discipline; or if any idea imbedded amid our associations is painful or turbulent, will eject it as summarily as a policeman an offender out of a crowd. So gentle is the action of Order, that it often introduces quiescence into the utmost turmoil of the mind, without any recognition of its presence.

In addition, then, to the function of Order exercised after Comparison and Causality, in giving breadth and generality to the conceptions of the mind, it has also a function antecedent to the ordinary action of the above faculties. In both cases by introducing a logical connexion over a large surface of facts or ideas, it tends to render them indelible in the mind, and so far usurps the proper functions of Memory. For it must be recollected that the corrosive element which chiefly destroys mental impressions is vagueness. Now, vagueness is caused either by excess of Association, implying the absence of intellect, or by the excess of intellectual action (chiefly characterized by Comparison and Causality), implying the absence of Association.

The conchologist minutely inspecting (*id est* comparing) the colours of a shell, may, in the very intensity of his gaze, miss his purpose of seeing definite outlines, by over eagerness. And the crystallographist, bending over the problem of the law of crystals, and using all his Causality to explore it, may defeat his purpose by not co-ordinating the laws of other sciences (such as mathematics) as assistants in his inquiry. In both such cases Order comes in to relieve the mind from its speciality of aim, and, by widening the prospect, to give security to our explorations. Order loves to place the inquirer upon the highest peak, from whence he may command the largest area of facts. And those gifted with this faculty are safe, if they remember that the surveyor, descending the mountain after his trigonometrical survey, has to fill it up in the most submissive reference to the meanest turnpike road in the valley. Yet, for the simple purpose of the retention in the mind of the features of the country, even though he did not follow it up, the traveller would find himself repaid. And with results equally happy will a man, deficient in the special faculty of Memory, introduce his faculty of Order to forge links, however artificial, among his ideas, by which each one will always have a rational bond of connexion with the rest.

By the use of the word artificial, I intend that it is most important for us not to confound the two occasions on which Order is called forth, the one early, the other late, in philosophical inquiry. Order, indeed, waits till a firm and solid foundation is laid before it ventures to rear its massive buttresses on high; but like a good architect, it is ever ready to assist its masons—Comparison and Causality—with a provisional scaffolding. From considering the work of Order, whether evinced in provisional generalizations or in the establishment of verified laws, we may estimate how powerfully it compensates for the absence of Memory. If, indeed, Order cannot, like Memory, reproduce and revivify past associations, and past forms, it can at least refer to its own work—those artistic combinations with which Order is ever clothing the nakedness of Fact.

On the one hand, the dominion of Memory over former facts is pure and simple. For whenever the simple succession of ideas, in their order of coming before the mind, is so indelibly impressed on the mind as to be capable of being recalled at will, either individually or in combination, THERE IS Memory. On the other hand, the dominion of Order over facts (represented of course by ideas), is over facts transmitted into, or forming a part of, generalizations, each portion of which involves the judicial action of the mind, and if remembered is remembered only in consequence of the impression produced on the mind by *mental acts*.

In conclusion, of the two faculties, Order and Memory, Order has, perhaps, the more dignity, as possessing most affinity with the other faculties concerned in inquiries after truth. But Memory seems to satisfy a more general necessity of the mind. Besides, Memory seems more independent of Order than Order is of Memory; for there must always be a moment of time during which we are compelled to retain a fact before any of the faculties which give the fact a philosophic shape can be used upon it; and this must be done by Memory, which can act "sponte suâ," and recal facts linked together by no bond; for to call mere succession a bond would be to assume the point in question. To the other compensation which Memory offers for want of Order, I will add, that Memory gives opportunity for the repeated examination of past facts or ideas, and can introduce, by the clearness of the images which it reproduces, the faculties of Comparison and Causality to give the last finish to the *details* which otherwise the faculty of Order would have given to the *whole.* On the other hand, Order compensates for the absence of Memory by suggesting new postures and assortments of old ideas, thus enabling the analytic action of Comparison and Causality to be applied from a new point of view. Thus some equality in the balance of results is produced.

Perhaps the majority of men would get on better if devoid of the assistance of Order, than if devoid of the assistance of Memory. For Order principally looks to large generalizations, which, from the nature of the case must be few as compared with the minute and practical details which, whether in speculation or action, it is left for the mass of mankind to carry out, in obedience to the expansive laws which genius discovers. For the details of action and of thought, habit produces the same facilities which, in large and expansive speculations, are achieved by a sense of Order and of Harmony. And we may note another divergence; that by the action of habit too prolonged men's minds become dead and automatic, and that by the action of the faculty of Order over-exercised the stability of the mind is imperilled by the vastness of the ideal landscape which it discloses.

To the mass of mankind Memory is of priceless importance. For no extrinsic aid could supply the void and uncertainty caused by the forgetfulness of particulars on which the safety of action mainly rests. On the other hand, to the philosopher a deficiency in his faculty of Order would be a far greater evil than a deficiency in his faculty of Memory.

For in the establishment of large and fruitful generalizations, the theory must long beforehand have been silently yet surely maturing from a constant verification of its various steps by reference to particular cases; and it is assumed that the processes of the intellect do stereotype themselves on the mind with a force which requires no assistance of Memory, which is chiefly required for those ideas between which there is no link forged by the intellect. Memory in general is chiefly applicable to those innumerable chains of facts and of ideas, between which we can discover no necessary connexion, but which we have to retain in the mind in combination as if they were necessarily connected.

What particulars, therefore, the philosopher needs, his faculty of Order would always point out, by description sufficient for him whose knowledge Order has arranged in marked receptacles, to which he may always refer. The whole of literature and science, as stored in books, act in fact as Memory to a philosopher gifted with the faculty of Order and who has duly exerted it. But if, on the other hand, the philosopher is strong in the accumulations which Memory heaps up of facts, but is deficient in the faculty of Order, then indeed rich in the raw material of knowledge he may be, but he will be skilless in that wisdom which can inspire dead matter with life and beauty; the diamonds may glitter in the sand, but where will be the hand which can arrange them in sparkling brilliancy around the coronet?

